# Systematic review of interventions to increase the provision of care for chronic disease risk behaviours in mental health settings: review protocol

**DOI:** 10.1186/s13643-018-0735-4

**Published:** 2018-04-30

**Authors:** Caitlin Fehily, Kate Bartlem, John Wiggers, Luke Wolfenden, Timothy Regan, Julia Dray, Jacqueline Bailey, Jenny Bowman

**Affiliations:** 1 The University of Newcastle, Callaghan, NSW Australia; 2 The Australian Prevention Partnership Centre (TAPPC), Sax Institute, Ultimo, NSW Australia; 3 Population Health, Hunter New England Local Health District, Wallsend, NSW Australia; 4 Hunter Medical Research Institute, Clinical Research Centre, New Lambton Heights, Australia

**Keywords:** Mental health, Physical health, Chronic disease, Mental health services, Service delivery, Risk behaviours

## Abstract

**Background:**

People with a mental illness experience a higher morbidity and mortality from chronic diseases relative to the general population. A higher prevalence of risk behaviours, including tobacco smoking, poor nutrition, harmful alcohol consumption and physical inactivity, is a substantial contributor to this health inequity. Clinical practice guidelines recommend that mental health services routinely provide care to their clients to address these risk behaviours. Such care may include the following elements: ask, assess, advise, assist and arrange (the ‘5As’), which has been demonstrated to be effective in reducing risk behaviours. Despite this potential, the provision of such care is reported to be low internationally and in Australia, and there is a need to identify effective strategies to increase care provision. The proposed review will examine the effectiveness of interventions which aimed to increase care provision (i.e. increase the proportion of clients receiving or clinicians providing the 5As) for the chronic disease risk behaviours of clients within the context of mental health service delivery.

**Methods:**

Eligible studies will be any quantitative study designs with a comparison group and which report on the effectiveness of an intervention strategy (including delivery arrangements, financial arrangements, governance arrangements and implementation strategies) to increase care provision specifically for chronic disease risk behaviours (tobacco smoking, poor nutrition, harmful alcohol consumption and physical inactivity). Screening for studies will be conducted across seven electronic databases: PsycINFO, MEDLINE, Excerpta Medica database (EMBASE), Psychology and Behavioural Sciences Collection, Scopus, Cochrane Central Register of Controlled Trials (CENTRAL) and Cumulative Index to Nursing and Allied Health Literature (CINAHL). Two authors will independently screen studies for eligibility and extract data from included studies. Where studies are sufficiently homogenous, meta-analysis will be performed. Where considerable heterogeneity exists (*I*^2^ ≥ 75), narrative synthesis will be used.

**Discussion:**

This review will be the first to synthesise evidence for the effectiveness of intervention approaches to facilitate care provision for chronic disease risk behaviours in the context of mental health service delivery. The results have the potential to inform the development of evidenced-based approaches to address the health inequities experienced by this population group.

**Systematic review registration:**

PROSPERO CRD42017074360.

**Electronic supplementary material:**

The online version of this article (10.1186/s13643-018-0735-4) contains supplementary material, which is available to authorized users.

## Background

In Australia, the gap in life expectancy for people with a mental illness is estimated to be 16 years for males and 12 years for females, with approximately 78% of this excess death being attributable to physical health conditions such as cardiovascular disease, diabetes and cancer [1]. A higher prevalence of modifiable risk behaviours including tobacco smoking, poor nutrition, physical inactivity and harmful alcohol consumption has been reported among people with a mental illness across multiple settings and psychiatric diagnoses, relative to the general population [2–4]. These risk behaviours contribute substantially to the higher morbidity and mortality from chronic diseases, and subsequently reduced life expectancy, experienced by this population group internationally [4–8].

The importance of assessing and managing the chronic disease risk behaviours of people with a mental illness within clinical practice is recognised in international best practice and clinical guidelines [2, 9, 10]. Moreover, it has been well recognised that there are opportunities to provide evidence-based preventive care for health risk behaviours systematically and routinely for a large proportion of persons with a mental illness within mental health care settings [11–13]. To address risk behaviours within clinical consultations generally, systematic review evidence [14–17] supports five recommended care elements in the ‘5As’ approach (ask, assess, advise, assist and arrange) (see Table 1 for definitions of the ‘5As’ elements), with some recent support also for an abbreviated ‘2As and an R’ model (ask, advice and refer), further recognising the need to manage time constraints and competing clinical priorities [18–20]. Provision of this care has been reported to be effective in reducing risk behaviours [21–24]; however, the provision of such care for chronic disease risks within mental health treatment settings is consistently reported to be sub-optimal in Australia [25–28] and internationally [29–31]. As such, there is a need to identify strategies to improve the delivery of this evidence-based care within mental health services.

**Table 1:** Definitions of the care elements in the ‘5As’ approach to preventive care provision [20, 51]

Care element	Definition
Ask	Asking clients about their current behaviour levels
Assess	Assessing readiness to change risk behaviours, and/or dependence (for tobacco smoking and alcohol consumption)
Advise	Advice to change behaviours or education around what constitutes risk, the individual’s level of risk, and/or guidelines for behaviours
Assist	Discussion of the benefits and barriers to change, providing counselling to change behaviours (such as motivational interviewing), and/or providing additional supports including pharmacotherapy, educational materials or self-help materials
Arrange	Referring the client to any health care provider or support service to support behaviour change (such as a telephone coaching service, dietician or support group).

Cochrane review evidence has suggested that a range of intervention strategies may increase adherence to clinical practice guidelines and policies generally, including electronic reminder systems [32, 33], education and training for staff [34], monitoring clinician behaviours and providing feedback [35] and coordination of care among multiple providers [36]. The Effective Practice and Organisation of Care (EPOC) taxonomy for health systems interventions identifies the following domains for categorising intervention strategies which aim to improve health care delivery: delivery arrangements (changes in who is responsible for care provision or how, when or where care is delivered), financial arrangements (changes in funding, insurance, purchasing of services and use of financial incentives), governance arrangements (changes in rules or processes such as policy changes) and implementation strategies (strategies to change the behaviour of healthcare organisations or clinicians, or the use of health services by clients) [37]. Although studies have shown such strategies to be effective in increasing provision of care specifically for chronic disease risk behaviours in general health settings [38–40], less is known about the effectiveness of these strategies within mental health service settings. The studies which have been conducted have trialled a range of intervention strategies [41] and have reported varied effectiveness. Strategies trialled with some effect in improving the provision of care for health risks in the context of mental health service delivery include delivery arrangements (such as additional specialist roles to support care provision [42, 43] and the co-location of mental and physical health services [44]) and implementation strategies (such as multi-strategy practice change interventions incorporating electronic reminder systems, staff education and training and clinician monitoring and feedback to encourage care provision with routine consultations of mental health services [45, 46]).

Two previous systematic reviews were identified that examined the effectiveness of intervention strategies to increase the delivery of physical health care in mental health settings [47, 48]. The first, a systematic review by Druss and von Esenwein [48], included studies of any design which aimed to improve linkage to and/or the quality of primary medical care for people with a mental illness, including screening, diagnosis and management of medical conditions (including hypertension, tuberculosis, sexually transmitted diseases and arthritis). A range of intervention approaches were identified, including training mental health staff to provide medical services, additional consultations with staff specifically to provide medical care and facilitated referrals to dedicated primary care services. Five of the six studies identified reported a statistically significant improvement in the number of appointments clients attended with a general medical provider following intervention. The second review, by Cerimele and Strain [47], reviewed interventions of any design which utilised one implementation strategy, placing a primary care provider in mental health settings, and identified four studies variously addressing biological (e.g. blood pressure weight and lipid screening) and behavioural chronic disease risks (e.g. tobacco smoking, poor nutrition, physical inactivity and substance use). From a narrative synthesis, the authors concluded that placing primary care providers in mental health settings may be effective in improving care provision (screening and counselling/advice), coordination of care with other health professionals and client health outcomes for biological (lipid screening, cancer screening, pap testing, blood pressure, and weight) and/or behavioural risks (tobacco smoking, substance use, nutrition, physical activity). Neither systematic review reported on the effectiveness of multiple intervention strategies in increasing the provision of care specifically for behavioural risks.

Given the absence of a systematic review synthesising the effectiveness of interventions in increasing care for behavioural chronic disease risks specifically within the context of mental health service delivery, and the range of intervention strategies [41] and varied effectiveness reported in individual studies to date, synthesis of the effectiveness of such intervention strategies is warranted.

### Objective

The aim of this review is to determine the effectiveness of interventions [37] designed to increase the provision of care (at least one component of the 5As) to address the chronic disease risk behaviours (tobacco smoking, poor nutrition, harmful alcohol consumption and physical inactivity) of clients within the context of mental health service delivery.

## Methods

All methods employed in the review will be consistent with the *Cochrane Handbook for Systematic Reviews of Interventions* [49]. This protocol has been reported in accordance with the Preferred Reporting Items for Systematic review and Meta-Analysis Protocols (PRISMA-P) [50] (see Additional file 1 for the populated PRISMA-P checklist).

### Eligibility criteria

#### Study design

Any quantitative study designs with a comparison group (usual care, no intervention or any alternative intervention) will be considered, such as randomised controlled trials including cluster randomised controlled trials, quasi-randomised trials and pre-post and interrupted time-series trials. Any studies without a comparison group will be excluded. There will be no restriction based on length of follow-up.

#### Participants

Services whose primary objective is to support the health and well-being of adults with a mental illness (i.e. predominantly over the age of 18) will be eligible. This may include three types of mental health services: bed-based (inpatient overnight acute residential care services); specialised community mental health care (including outpatient services such as community mental health services, clinical psychologists and private psychiatrists); and community-based mental health support services (non-clinical mental health services that support people with a mental illness to live independently in the community, including residential respite care, group and individual support and rehabilitation services, non-governmental mental health organisations and community managed organisations). Settings exclusively providing care for substance use will be excluded.

#### Interventions

To be eligible, studies must have aimed to increase the delivery of at least one preventive care element (ask, assess, advise, assist and arrange) for at least one of the four key chronic disease risk behaviours (tobacco smoking, poor nutrition, harmful alcohol consumption and/or physical inactivity). In order to be considered, the intervention must have aimed to increase the delivery of care within the context of mental health care delivery, by staff or clinicians of the service. Studies where research staff provide the care will be excluded.

All types of intervention strategies will be considered. These include, but are not limited to, delivery arrangements (such as additional personnel to support the provision of care), financial arrangements (such as changes to funding and insurance schemes), governance arrangements (such as policy changes), and implementation strategies (such as audit and feedback, electronic tools and education and training for staff) [37]. Interventions may be singular or multi-component.

#### Primary outcomes

Studies will be included if they quantitatively assess the provision or receipt of at least one care element (ask, assess, advice, assist and arrange) for at least one chronic disease risk behaviour (tobacco smoking, poor nutrition, harmful alcohol consumption or physical inactivity) in the context of mental health service delivery. Use of the 5As terminology for the elements of care will not be required and will be inferred by the extractor based on definitions [20, 51] of the 5As [52]. Broadly, *ask* will be asking clients about their current behaviour levels for at least one of the risk behaviours. *Assessment* will be considered assessing readiness to change and/or dependence (for tobacco smoking and alcohol consumption). *Advice* will include any type of advice to change behaviours or education around the individual’s level of risk, the definitions of risk and/or guidelines for behaviours. *Assist* may include discussing the benefits of and barriers to change, providing counselling to change behaviours such as motivational interviewing and/or providing additional supports such as pharmacotherapy or educational materials. *Arrange* will include making a referral to any health care provider or support service to address behaviour change such as a telephone support service, dietician or support group [20, 51]. How the risk behaviours are operationalised will be dependent on how each study defines such behaviours. For instance, poor nutrition may include markers such as daily energy intake, sodium intake, saturated fat intake or insufficient fruit and vegetable consumption.

Outcomes can be reported as absolute care provision or receipt (e.g. the percentage of clients provided an element of care before and after an intervention) or a relative change in the provision or receipt of care. Data may be derived from a variety of sources such as client report, clinician report, medical record audit or administrative records. For studies reporting multiple follow-up assessments, data will be extracted for the final follow-up point.

Outcome data that reports the provision or receipt of care for an individual behavioural risk, or combined with multiple behavioural risks will be extracted. Where outcome data is combined for multiple risks that includes both behavioural and non-behavioural risks (e.g. tobacco smoking and blood pressure) and the impact of the intervention on behavioural risks cannot be extracted separately to non-behavioural risks, that data will not be extracted. Corresponding authors will be contacted to determine if any data for individual risk behaviours can be provided.

#### Secondary outcomes

1. Measures of client risk behaviours including tobacco smoking, nutrition, alcohol consumption and physical activity levels. These measures could be collected from a variety of sources such as client report, clinician report following an assessment, medical records, observation and biochemical measures.

2. Any estimate of the costs and/or cost effectiveness of intervention strategies to improve the delivery of care for chronic disease risk behaviours provided in mental health settings.

#### Publication characteristics

There will be no exclusion criteria based on the country where a study was undertaken. Included studies must be published in English and have been published from 1998 to present. Given that implementation science is a relatively new field, this timeframe is sufficient to capture all relevant research.

### Information sources

#### Electronic databases

The following electronic databases will be searched: PsycINFO, MEDLINE, Excerpta Medica database (EMBASE), Psychology and Behavioural Sciences Collection, Scopus, Cochrane Central Register of Controlled Trials (CENTRAL) and Cumulative Index to Nursing and Allied Health Literature (CINAHL).

#### Other sources

Hand searches will be conducted on the reference lists of included studies: the first 200 citations of Google Scholar and four relevant journals in the field from the past 3 years (Psychiatric Services, Implementation Science, British Journal of Psychiatry Bulletin and BMC Health Services Research). Experts in the field will also be consulted for additional references. Authors of included studies will be contacted to check for further related publications and potential studies.

### Search strategy

The search strategy will include terms for (a) the study setting (e.g. mental health service, psychosocial support service, community mental health), (b) the four risk behaviours (tobacco smoking, poor nutrition, physical inactivity and harmful alcohol consumption), and (c) the study type (intervention or implementation studies). Search filters will be included for mental health service types and risk behaviours that were used in other, similar systematic reviews [52, 53]. Search terms for study type (interventions and implementation studies) will be adapted from a glossary for dissemination and implementation research [54] and similar systematic reviews [55]. The search strategy will be adapted for each database as required (see Additional file 2 for the draft search strategy for MEDLINE).

### Study records

#### Data management

EndNote will be used to remove duplicates, to assist in obtaining full-text papers and to store and manage review records. RevMan software will be used for pooling of trial data and meta-analyses.

#### Selection of studies

Duplicate articles will be removed. Two reviewers will independently assess the titles and abstracts of studies identified using the above search strategy to determine their eligibility based on the inclusion criteria. The reviewers will not be blinded to author name, author study institution or journal title. Articles that do not meet inclusion criteria will be excluded. The full texts of the remaining papers will be obtained and assessed independently by two reviewers to determine study eligibility. Any disagreement between the two reviewers regarding study eligibility will be resolved via consensus or, if required, a third reviewer. Where there is no sufficient study details, corresponding authors will be contacted for further details to determine study eligibility. If sufficient information remains unavailable, the study will be deemed ineligible.

#### Data collection process

Two study reviewers will independently extract data for each included study using a standardised form which will be piloted. Disagreements regarding data extraction will be resolved through consensus between the two authors or by a third author where discrepancies remain unresolved. Where there is insufficient data reported for primary outcomes, corresponding authors will be contacted for clarification. One review author will transcribe data from eligible studies into RevMan software using data extraction forms, and a second author will check this process [56–58]. The following information will be extracted:

- Author and year of publication, study design, mental health service type, country and participant/service demographics.
- Characteristics of the intervention including intervention and comparison group conditions, intervention duration and intensity, type of intervention [37], strategies implemented, who delivered the intervention (e.g. all staff, select staff or a single staff member), target of the intervention (who was expected to provide the care), care element(s), health behaviour(s) addressed, any policy that the mental health service had with regard to chronic disease behaviour care and measures related to intervention fidelity.
- Data pertaining to primary and secondary outcomes including data source/collection method, data collection time point, effect size and measures of outcome variability.
- Information required for assessment of potential study bias (see Assessment of risk of bias).

#### Assessment of risk of bias

For randomised controlled trials, risk of bias for each included study will be assessed independently by two review authors against the Cochrane Handbook for Systematic Reviews of Interventions study characteristics including selection bias (sequence generation and allocation concealment), performance bias (blinding of participants and personnel), detection bias (blinding of outcome assessment), attrition bias (incomplete outcome data), reporting bias (selective reporting) and other potential sources of bias [49]. For non-randomised controlled trials, potential confounding will be assessed [49]. Risk of bias for cluster-randomised trials will be assessed against additional criteria, including recruitment to cluster, baseline imbalance, loss of clusters and incorrect analysis [49]. Any additional biases specific to individual study designs will be assessed by the reviewers and reported.

### Data analysis

Where studies are sufficiently homogenous (*I*^2^ < 75%; chi-square *p* > 0.1), a random effects meta-analysis will be performed for each element of care (ask, assess, advise, assist and arrange) by the four risk behaviours (tobacco smoking, poor nutrition, harmful alcohol consumption and physical inactivity). Continuous outcomes will be pooled and reported as a mean difference where consistent measures are used or a standardised mean difference where different measures are used to report a comparable outcome. Binary outcomes will be pooled and effect estimate reported using odds ratios. Where possible, sub-group analyses will be conducted for different intervention strategies, mental health service type and the four risk behaviours. A sensitivity analysis will be conducted to exclude studies which are categorised as high risk of bias. Where studies are not sufficiently homogenous, trial outcomes will be described narratively.

#### Assessment of study heterogeneity

Heterogeneity will be assessed via visual inspection of forest plots and consideration of the *I*^2^ statistic. Where considerable heterogeneity is found (*I*^2^ ≥ 75) [49], the sources of heterogeneity will be investigated through sub-group analysis on study setting (mental health service type), design, outcomes and interventions.

#### Issues of clustering

For any included cluster randomised controlled trials, adjustments will be made for unit of analysis error by applying intraclass correlation values. If not reported, intraclass correlations will be requested from corresponding authors, and if they remain unavailable, estimates from similar studies will be used to adjust for clustering [49].

### Assessment of reporting bias

Funnel plots will be used to determine possible reporting bias in included studies.

### Confidence in cumulative evidence

The strength of the body of evidence will be assessed using the GRADE approach developed by the Grades of Recommendation, Assessment, Development and Evaluation Working Groups [59]. This approach includes assessment of each individual outcome per trial across five key areas: risk of bias within included studies (methodological quality), directness of evidence (relevance to the review question), heterogeneity (inconsistency), precision of effect estimates, and risk of publication bias.

### Ethics and dissemination

Ethics approval is not required. The findings of this review will be disseminated via publication of the final review manuscript and conference presentations.

## Discussion

This systematic review will be the first to examine the effectiveness of interventions to increase care provision for chronic disease risk behaviours in mental health settings. The high prevalence of chronic disease risk behaviours among persons with a mental illness is contrasted with a low prevalence of care. An effective intervention approach to facilitate the delivery of care for chronic disease risk behaviours within the context of mental health service delivery has the potential to reduce their high prevalence and consequently reduce the chronic disease burden experienced by persons with a mental illness. The synthesis of evidence in this review will provide clarity around the effectiveness of such interventions. The findings hold the potential to translate into effective implementation strategies to improve the quality of care for reducing health risk behaviours among clients of mental health services.

## Additional files

Additional file 1: PRISMA-P 2015 populated Checklist. (DOCX 23 kb)

Additional file 2: Draft search strategy for MEDLINE. (DOCX 28 kb)

## Abbreviations

2As and an R: Assess, advise and refer
5As: Ask, assess, advise, assist and arrange
CENTRAL: Cochrane Central Register of Controlled Trials
CINAHL: Cumulative Index to Nursing and Allied Health Literature
EMBASE: Excerpta Medica database
