# Supplementary material for: Systematic review of interventions to increase the provision of care for chronic disease risk behaviours in mental health settings: review protocol
Source: Syst Rev. 2018 Apr 30;7:67. doi: 10.1186/s13643-018-0735-4 (PMC5928577; doi:10.1186/s13643-018-0735-4)
Supplement: Supplementary file 2 — Draft search strategy for MEDLINE. (DOCX 28 kb) [file 13643_2018_735_MOESM2_ESM.docx]

# Additional file 2 – DRAFT SEARCH STRATEGY FOR MEDLINE

Database(s): **Ovid MEDLINE(R) Epub Ahead of Print, In-Process & Other Non-Indexed Citations, Ovid MEDLINE(R) Daily and Ovid MEDLINE(R)**1946 to Present

Search Strategy:

| **#** | **Searches** | **Results** |
| --- | --- | --- |
| 1 | mental health services/ or community mental health services/ or emergency services, psychiatric/ or social work, psychiatric/ | 50657 |
| 2 | community psychiatry/ or preventive psychiatry/ | 1947 |
| 3 | Hospitals, Psychiatric/ | 24296 |
| 4 | Community Mental Health Centers/ | 2820 |
| 5 | (inpatients/ or outpatients/) and (mental health or mental illness* or psychiatric or mental disorder*).mp. | 5887 |
| 6 | ((psychiatric or mental health) adj3 (centre* or center* or service* or hospital* or clinic* or ogani?ation*)).tw. | 47084 |
| 7 | 1 or 2 or 3 or 4 or 5 or 6 | 104225 |
| 8 | "tobacco use cessation"/ or smoking cessation/ | 26129 |
| 9 | Smoking/ | 131357 |
| 10 | smoking.tw. | 193132 |
| 11 | nutrition*.mp. | 336002 |
| 12 | Fruit/ | 36485 |
| 13 | Vegetables/ | 21552 |
| 14 | Diet/ | 141458 |
| 15 | Obesity/ or Overweight/ or Body Weight/ | 325962 |
| 16 | (fruit* or vegetables* or diet* or obes* or overweight).tw. | 773173 |
| 17 | drinking behaviour/ or alcohol drinking/ | 60666 |
| 18 | binge drinking/ | 1212 |
| 19 | (drinking or alcohol*).tw. | 355803 |
| 20 | exercise/ | 89694 |
| 21 | physical fitness/ | 25351 |
| 22 | sedentary lifestyle/ | 6518 |
| 23 | (physical activit* or exercise or physical fitness or physical inactivit* or sedentary).tw. | 308198 |
| 24 | smoke-free policy/ | 588 |
| 25 | chronic disease/ | 246720 |
| 26 | Cardiovascular Diseases/ | 128995 |
| 27 | (cardiovascular adj3 (disease* or risk*)).tw. | 197159 |
| 28 | (lifestyle* adj3 (factor or behavior* or behaviour*)).tw. | 4853 |
| 29 | (risk adj3 (behavior* or behaviour* or factor*)).tw. | 520597 |
| 30 | 8 or 9 or 10 or 11 or 12 or 13 or 14 or 15 or 16 or 17 or 18 or 19 or 20 or 21 or 22 or 23 or 24 or 25 or 26 or 27 or 28 or 29 | 2648113 |
| 31 | (intervention* or therap* or program* or form* or treatment* or training* or educat* or innovation* or practice* or polic* or feedback or prompt* or reminder* or tool* or effectiveness or (system* adj2 change*) or implement* or dissemin* or translat* or organi?ational change or incentive* or audit* or integrat*).tw. | 10182810 |
| 32 | (specialist or ((addition* or extra or increase*) adj3 (staff* or clinician* or role* or personnel))).tw. | 70563 |
| 33 | 31 or 32 | 10205712 |
| 34 | 7 and 30 and 33 | 7728 |
| 35 | limit 34 to (english language and yr="1998-Current") | 5309 |
| 36 | animals/ not (humans/ and animals/) | 4403503 |
| 37 | 35 not 36 | 5304 |
